# Vascular Phenotypes and Recurrent Arterial Events After Nontraumatic Cervical Artery Dissection

**DOI:** 10.1016/j.jacadv.2026.103432

**Published:** 2026-11-05

**Authors:** Daniella Kadian-Dodov, Aleksan Khachatryan, Alan Weinberg, Amy R. Kontorovich, Jason C. Kovacic, Jeffrey W. Olin

**Affiliations:** a Mount Sinai Fuster Heart Hospital, Icahn School of Medicine at Mount Sinai, New York, New York, USA; b Department of Population Health Science and Policy, Icahn School of Medicine at Mount Sinai, New York, New York, USA; c Cardiovascular Research Institute, Icahn School of Medicine at Mount Sinai, New York, New York, USA; d Victor Chang Cardiac Research Institute, Darlinghurst, Australia; e St. Vincent’s Clinical School, University of NSW, Australia

**Keywords:** aneurysm, arteriopathy, carotid artery dissection, fibromuscular dysplasia, vertebral artery dissection

## Abstract

**Background:**

Nontraumatic cervical artery dissection (CeAD) is often approached as a localized cervicocranial vascular disorder, although overlap with fibromuscular dysplasia (FMD) and other arteriopathies suggests broader vascular disease.

**Objectives:**

The objectives of the study was to determine whether head-to-pelvis imaging identifies clinically meaningful vascular phenotypes after nontraumatic CeAD and whether these phenotypes are associated with recurrent arterial events.

**Methods:**

Consecutive adults with CeAD evaluated in an institutional vascular medicine clinic underwent head-to-pelvis computed tomography and/or magnetic resonance angiography. Patients were classified as isolated CeAD, FMD-associated CeAD (FMD-CeAD), or multiple artery aneurysm and dissection without FMD (MAAD). Outcomes were recurrent CeAD and incident extracervical artery dissection.

**Results:**

Among 315 patients, 136 (43.2%) had isolated CeAD, 100 (31.7%) had FMD-CeAD, and 79 (25.1%) had MAAD-CeAD. Overall, 179 patients (56.8%) had systemic vascular abnormalities, and 125 (39.7%) had findings below the neck. Recurrent CeAD was most frequent in isolated CeAD. Incident extracervical artery dissection occurred most frequently in MAAD-CeAD. Isolated CeAD was associated with recurrent CeAD compared with FMD-CeAD (HR: 3.26; 95% CI: 1.43 to 7.43). Among patients with FMD-CeAD or MAAD-CeAD, MAAD-CeAD was associated with incident extracervical artery dissection after adjustment for age at index CeAD (HR: 3.75; 95% CI: 1.17-12.00; *P* = 0.026), with similar results after additional adjustment for baseline aneurysm.

**Conclusions:**

Head-to-pelvis imaging identified frequent extracervical vascular abnormalities in patients with CeAD and phenotypes with distinct recurrent-event patterns. These findings support a cardiovascular arteriopathy framework for CeAD and provide a rationale for prospective studies of phenotype-guided imaging, genetic evaluation, counseling, and surveillance.

## Background

Spontaneous extracranial cervical artery dissection (CeAD) (internal carotid or vertebral arteries) accounts for approximately 20% of cerebrovascular events in patients ≤45 years.^1^ Among patients with any CeAD, up to 20% have multiple CeAD, with long-term recurrence rates estimated at 1% per year for at least 10 years after the incident event.^2^, ^3^ The pathogenesis of CeAD is incompletely understood, but an underlying systemic arteriopathy is supported by frequently observed arterial tortuosity, as well as an identified shared genetic locus with fibromuscular dysplasia (FMD) and spontaneous coronary artery dissection (SCAD).^4^, ^5^, ^6^, ^7^ Clinical overlap between these entities is increasingly appreciated, with concomitant rates of CeAD reported in 15% to 39% of patients with FMD.^1^, ^3^, ^8^, ^9^, ^10^

Expert consensus recommends that every patient with FMD or SCAD undergo one-time head-to-pelvis imaging (preferably with computed tomography angiography (CTA) due to better image resolution) to identify the presence of FMD, aneurysm(s), and/or dissections in other arterial beds.^8^, ^11^, ^12^ Patients with CeAD have also been found to have clinically relevant findings from cross-sectional imaging beyond the head and neck,^9^, ^13^ yet universal imaging for this population has not yet been included in CeAD expert consensus statements.^14^ It remains uncertain whether CeAD should be approached as an isolated cervicocranial event or as a manifestation of a broader arteriopathy identifiable by systematic vascular imaging. We therefore sought to determine whether comprehensive head-to-pelvis vascular imaging after nontraumatic CeAD identifies distinct systemic vascular phenotypes and whether these phenotypes are associated with recurrent cervical and extracervical artery events.

## Methods

### Study population

Epic SlicerDicer queries were implemented to identify patients with CeAD evaluated in a single-center vascular medicine clinic between January 2016 and December 2024. Search query terms included International Classification of Diseases-10 codes I77.71 and I77.74 as a diagnosis or listed medical history. Clinical outcomes were ascertained longitudinally through structured follow-up and clinical imaging. The diagnoses of FMD and/or CeAD were confirmed by a board-certified vascular medicine specialist (D.K.D. or J.W.O.), who evaluated all patients and personally reviewed all of the imaging (Figure 1). Patients included both individuals diagnosed at our institution and patients referred after outside diagnosis for vascular medicine evaluation.

**Figure 1:**
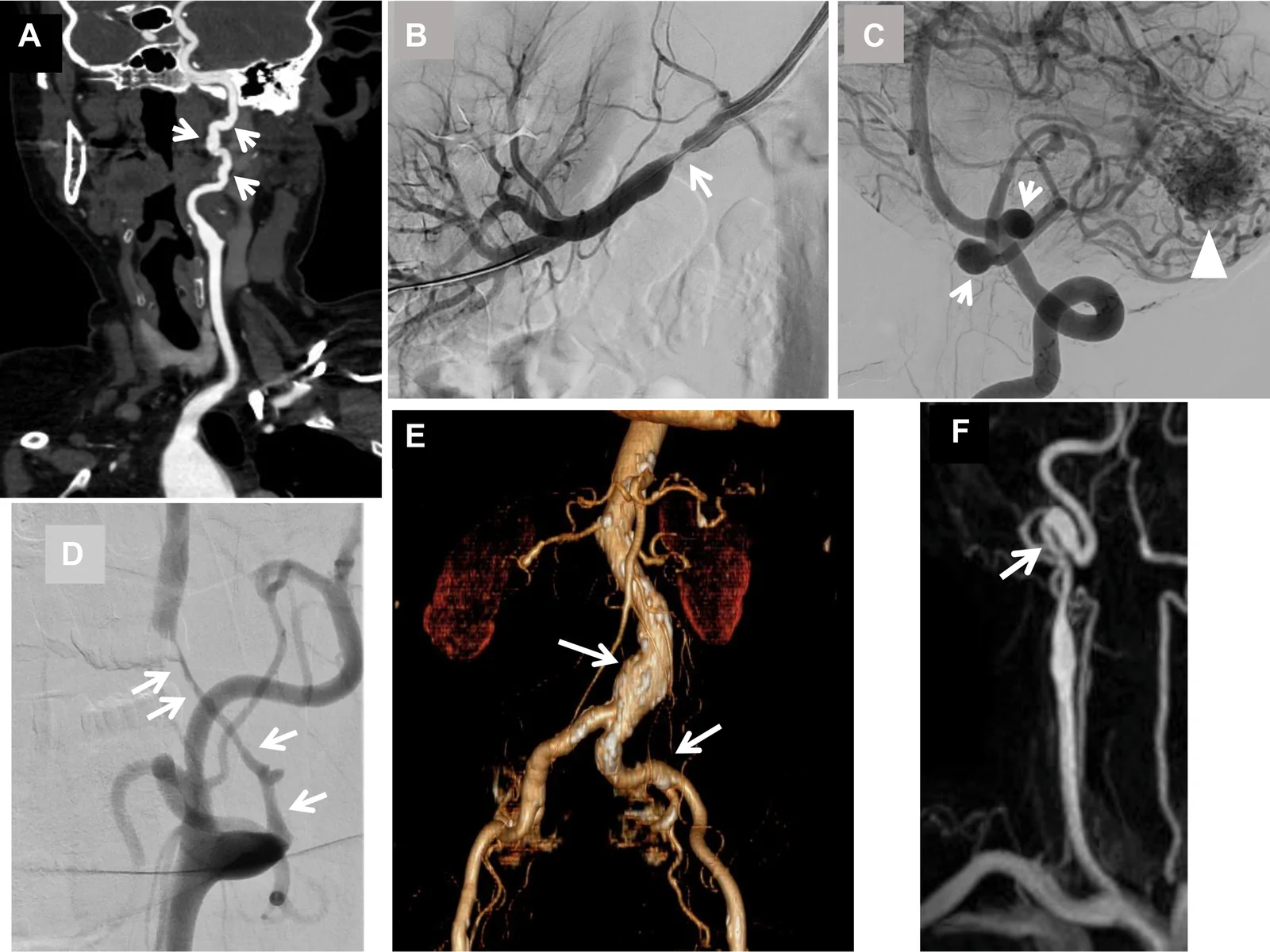
**Characteristics of Fibromuscular Dysplasia** (A) Computed tomography angiogram demonstrating tortuosity and multifocal fibromuscular dysplasia of the mid-distal right internal carotid artery (arrows), (B) catheter angiogram showing focal fibromuscular dysplasia of the right renal artery (arrow), (C) 2 aneurysms of the distal left vertebral artery (arrows) and a cerebellar ateriovenous malformation (arrowhead) visualized on catheter-based angiography, (D) dissection of the right internal carotid artery on catheter-based angiography demonstrating severe stenosis (string sign) (arrows) with return to normal caliber vessel distally, (E) computed tomography angiogram (volume rendered) demonstrating tortuosity of the aorta and iliac arteries and (F) internal carotid artery showing extreme tortuosity (S-curve) and ectasia.

### Inclusion and exclusion criteria

Patients at least 18 years with a confirmed diagnosis of nontraumatic CeAD on imaging, ≥2 clinical visits, and with complete head-to-pelvis cross-sectional imaging available for review were included. Isolated CeAD was defined as CeAD without any other arterial abnormality noted at the time of initial evaluation with pan-vascular imaging. CeAD was confirmed on imaging if at least 1 of the defined manifestations was observed (Figure 2). Patients with vasculitis, atherosclerosis-associated dissection, or iatrogenic dissection were excluded. The diagnosis of FMD was made following the recommendations in the International Consensus on the Diagnosis and Management of Fibromuscular Dysplasia^10^ and the American Heart Association State of the Science manuscript on FMD.^15^ As such, patients had to have 1 of 2 angiographic appearances: 1) multifocal FMD—alternating areas of stenosis and dilatation (“string of beads”) in any arterial bed except for the aorta or coronary arteries; and 2) focal FMD—a concentric or tubular stenosis generally in the renal or carotid arteries (Figure 1). In a patient with multifocal or focal FMD, the presence of aneurysms, dissections, tortuosity, and ectasia are considered to be manifestations of FMD.^11^, ^13^, ^16^ Patients with a carotid or vertebral artery dissection but without either of the aforementioned angiographic appearances do not have FMD.^11^, ^16^ Multiple artery aneurysm and dissection (MAAD) associated CeAD was diagnosed as the presence of aneurysm or dissection in another vascular site, below the head and neck, as well as the absence of FMD. The Mount Sinai Institutional Review Board approved this research (IRB# 19-00104).

**Figure 2:**
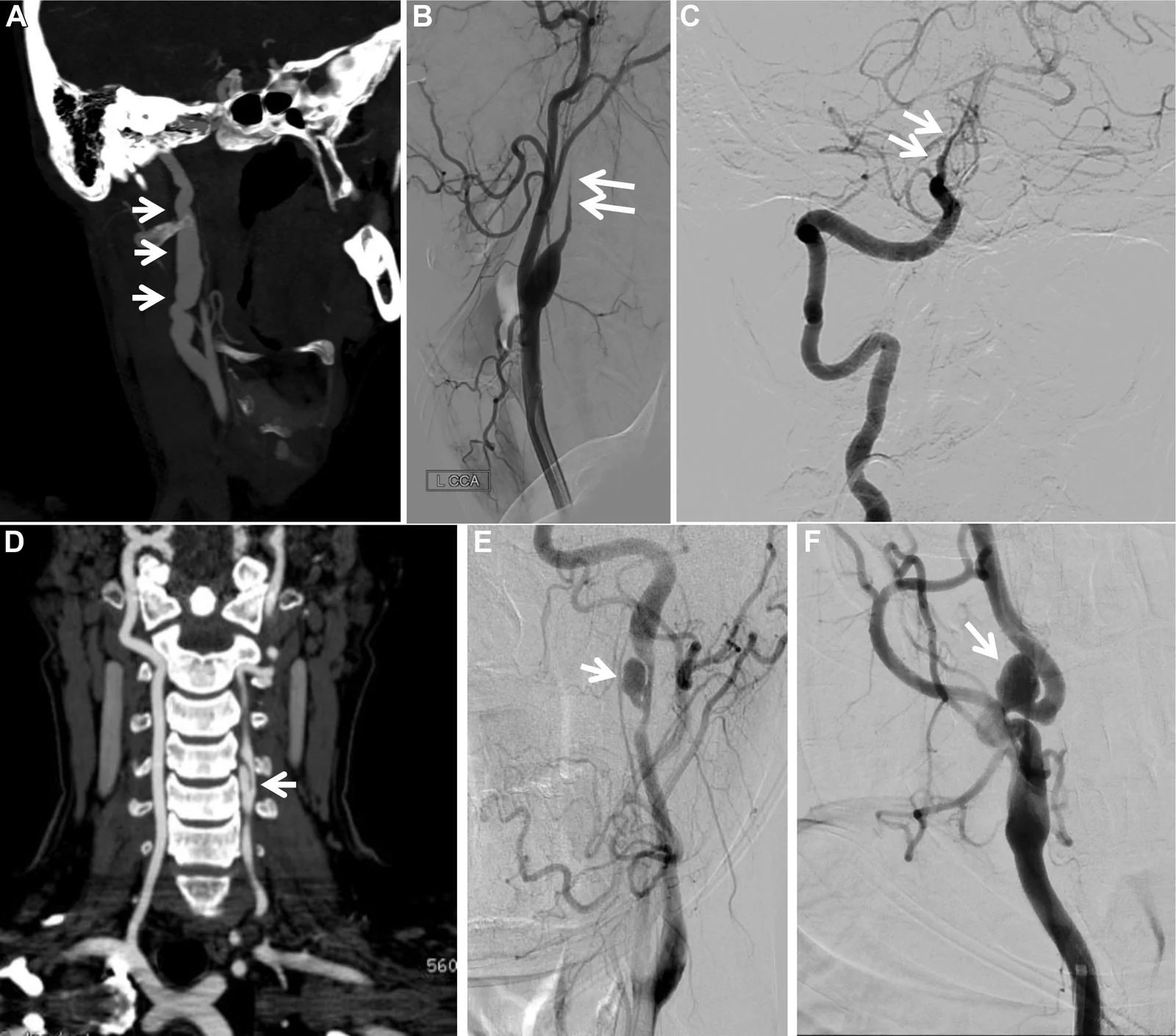
**Imaging Findings of Cervical Artery Dissection** (A) Computed tomography angiogram demonstrating diffuse irregularity and ectasia typical of vessel remodeling after dissection (arrows), (B) catheter-based angiography demonstrating tapering of the proximal internal carotid artery with flame-tipped occlusion typical of a carotid artery dissection (arrow), (C) distal stenosis due to dissection of the right vertebral artery extending to the basilar artery (arrow), (D) intimal flap with double lumen seen in the left vertebral artery on computed tomography angiogram (arrow), (E) dissection resulting in pseudoaneurysm of the left internal carotid artery (arrow), and (F) broad-based pseudoaneurysm in an area of prior dissection of the right internal carotid artery seen on catheter-based angiography.

### Data collection

This study is reported in accordance with the Strengthening the Reporting of Observational Studies in Epidemiology (STROBE) guidelines. Baseline and clinical information were collected for each patient, including: age at diagnosis of FMD and/or CeAD, sex, race, hypertension (defined as the need for antihypertensive agent(s)—already on or prescribed—at the initial visit), history of smoking, substance use (former or current use recorded in social history or positive urine toxicology), family history (1st or 2nd degree relative with aneurysm, dissection, FMD, cardiovascular events ≤50 years, and sudden death), type and location of FMD (aneurysm, dissection, and/or ectasia in addition to the presence of pathognomonic focal/multifocal disease), location and size of cerebral aneurysms, location of noncerebral aneurysms, location and characteristics of CeAD (associated aneurysm and pseudoaneurysm), and the location of the non-CeAD dissection. Available head-to-pelvis imaging with CTA, magnetic resonance angiography (MRA), or catheter-based angiography was reviewed by 2 investigators (D.K.D. and J.W.O.). The locations of FMD, arterial dissection, aneurysm, as well as vascular events (stroke, transient ischemic attack, intervention, or repeat dissection identified in clinical notes and confirmed with imaging) were recorded and adjudicated by D.K.D. and J.W.O.

It has been our policy to obtain head-to-pelvis imaging on all FMD patients evaluated in our clinic since 2014. We instituted the same policy in 2016 for patients presenting with nontraumatic CeAD at our center. Radiologic follow-up after index CeAD was recommended by vascular medicine physicians (J.W.O. and D.K.D.) and adapted to the initial clinical presentation. Generally, all CeAD patients underwent repeat CTA or MRA head and neck with clinical follow-up at 6 months, 12 months, and then annually. Following the index extracervical imaging with CTA, repeat imaging was obtained based on findings at initial scan (eg monitoring of aneurysm size or dissection) or for new clinical symptoms. The classification of patients into the 3 phenotype groups was based on index imaging; subsequent vascular findings or events were reported according to the index phenotype, and no patients were reclassified during follow-up. Missing data were infrequent and handled using complete-case analysis.

Genetic testing was performed as part of clinical care in selected patients at the discretion of the treating vascular medicine and/or cardiovascular genetics team. Testing generally consisted of commercially available multigene panels for heritable thoracic aortic disease, connective tissue disorders, and arterial aneurysm/dissection syndromes. Results were categorized as pathogenic/likely pathogenic, variant of uncertain significance (VUS), or negative according to the clinical laboratory report. Genetic testing was not performed systematically across the cohort.

### Statistical analysis

Baseline characteristics were reported as median (IQR), or as mean ± SD for normally distributed continuous variables. Categorical variables were summarized as counts and percentages. Continuous variables were compared using analysis of variance or Kruskal-Wallis tests, as appropriate. Categorical variables were compared using chi-square or Fisher exact tests. Time-to-event analyses were performed using Kaplan-Meier methods and Cox proportional hazards models, with follow-up beginning at the index CeAD. Models evaluating recurrent CeAD included all 3 imaging-defined phenotypes and were adjusted for age at CeAD diagnosis, sex, hypertension, and baseline aneurysm presence. For incident extracervical artery dissection, Firth penalized Cox regression was used because of the limited number of outcome events. The multivariable model was adjusted for age at the index CeAD. Because aneurysm is also a component of the imaging-defined vascular phenotype, a sensitivity model additionally adjusted for baseline aneurysm. All analyses were performed with SAS 9.4 (SAS Institute Inc).

## Results

### Study population and vascular phenotype classification

We identified 366 patients with CeAD, of whom 51 were excluded due to incidental CeAD identified in the context of a known heritable arteriopathy or absent complete head-to-pelvis imaging. The final cohort consisted of 315 patients who underwent comprehensive vascular imaging. On head-to-pelvis imaging with CTA or MRA, 179 of 315 patients (56.8%) had evidence of systemic vascular abnormalities, including FMD, aneurysm, or dissection. When assessing the utility of chest/abdomen/pelvis imaging, 125 of 315 patients (39.7%) had systemic vascular findings below the neck. Patients were classified into 3 imaging phenotypes (Central Illustration): isolated CeAD (n = 136, 43.2%), FMD-CeAD (n = 100, 31.7%), or MAAD-CeAD (n = 79, 25.1%) (Table 1).

**Table 1:** Baseline Characteristics

	Isolated CeAD (n = 136)	FMD-CeAD (n = 100)	MAAD-CeAD (n = 79)	*P* Value
Age at diagnosis of CeAD (y), mean ± SD	42.1 ± 11.9	51.5 ± 12.2	48.4 ± 11.7	<0.001
Female, n (%)	100 (73.5)	90 (90)	46 (58.2)	<0.001
Race, n (%)				0.111
Caucasian	101 (74.2)	81 (81)	62 (78.5)	
Asian	2 (1.5)	2 (2)	6 (7.6)	
Black	7 (5.2)	3 (3)	4 (5.1)	
Unknown	26 (19.1)	14 (14)	7 (8.8)	
Hypertension, n (%)	36 (26.5)	44 (44)	42 (53.2)	<0.001
Active or former smoker, n (%)	27 (19.8)	25 (25)	13 (16.5)	0.371
Active or former drug use, n (%)	6 (4.4)	1 (1)	3 (3.8)	0.348
Marijuana	6	1	2	
Cocaine	0	0	1	
Family History of 1st/2nd degree relative, n (%)				
FMD	4 (2.9)	3 (3)	0	0.332
Aneurysm	25 (18.4)	16 (16)	9 (11.4)	0.420
Dissection	16 (11.8)	8 (8)	8 (10.1)	0.664
Sudden death	11 (8.1)	8 (8)	10 (12.6)	0.492
MI/stroke age <50 years	21 (15.4)	22 (22)	16 (20.2)	0.397
Location of CeAD, n (%)				0.626
Carotid	71 (52.2)	61 (61)	44 (55.7)	
Vertebral	48 (35.3)	27 (27)	23 (29.1)	
Both	17 (12.5)	12 (12)	12 (15.2)	
Number of cervical arteries dissected at index event, n (%)				0.688
1	86 (63.2)	71 (71)	47 (59.4)	
2	40 (29.4)	24 (24)	27 (34.2)	
3	6 (4.4)	4 (4)	4 (5.1)	
4	4 (2.9)	1 (1)	1 (1.3)	
Presenting signs/symptoms at CeAD diagnosis, n (%)^a^				
Asymptomatic	2 (1.5)	3 (3)	5 (6.3)	0.146
Pulsatile tinnitus	11 (8.1)	16 (16)	9 (11.4)	0.168
Head or neck pain	95 (69.8)	61 (61)	46 (58.2)	0.169
Transient ischemic attack	43 (31.6)	28 (28)	23 (29.1)	0.824
Horner syndrome	15 (11)	8 (8)	5 (6.3)	0.471
Stroke	47 (34.6)	21 (21)	23 (29.1)	0.076
CeAD-related pseudoaneurysm, n (%)				
Carotid	33 (24.3)	28 (28)	22 (28)	0.760
Vertebral	8 (5.9)	13 (13)	4 (5.1)	0.102
CeAD-related intervention^a^, n (%)	16 (11.8)	10 (10)	11 (13.9)	0.720
Mechanical thrombectomy	6	0	0	
Thrombolysis	4	3	3	
Endovascular angioplasty and/or stent	11	2	3	
Endovascular repair of aneurysm/pseudoaneurysm	3	6	5	
Surgical repair of aneurysm	0	0	0	
Craniectomy	0	0	1	
Other	0	1	0	

CeAD = cervical artery dissection; FMD = fibromuscular dysplasia; MAAD = multiple artery aneurysm and dissection; MI = myocardial infarction.

a Interventions are not mutually exclusive.

### Baseline clinical characteristics across phenotypes

Patients with isolated CeAD were younger than those with FMD-CeAD or MAAD-CeAD. The proportion of female patients differed across phenotypes, with the highest proportion in FMD-CeAD and the lowest in MAAD-CeAD (*P* < 0.001). Index CeAD characteristics were generally similar across phenotypes (Table 1). Hypertension was more common in patients with FMD-CeAD and MAAD-CeAD.

### Extracervical aneurysm, dissection, and arterial bed involvement

Among patients with FMD-CeAD, most had multifocal FMD (99%) and multivessel involvement (>1 arterial bed) (Table 2). Aneurysms were identified in 39% of patients with FMD-CeAD as compared to 71% of patients with MAAD-CeAD (*P* < 0.001), with distinct distributions across vascular beds (Table 2). In MAAD-CeAD, thoracic aortic aneurysm was the most common location (30.3%), whereas aneurysms in FMD-CeAD most frequently involved the cerebral, mesenteric, and renal arteries. Extracervical artery dissection was identified in 12% of patients with FMD-CeAD and 46.8% of patients with MAAD-CeAD (*P* < 0.001), most commonly affecting the mesenteric and renal arteries in the MAAD-CeAD group. Aortic arch anomalies did not differ significantly among phenotypes, including bovine arch configuration in 24 patients with isolated CeAD, 27 with FMD-CeAD, and 16 with MAAD-CeAD (*P* = 0.210); direct origin of the left vertebral artery in 8, 7, and 4 patients, respectively (*P* = 0.867); and aberrant right subclavian artery in 4, 0, and 3 patients, respectively (*P* = 0.130).

**Table 2:** Systemic Arterial Findings in Patients With FMD-CeAD Versus MAAD-CeAD

	FMD-CeAD (n = 100)	MAAD-CeAD (n = 79)	*P* Value
Aneurysm^a^^,^^b^, n (%)	39 (39)	56 (71)	<0.001
Cerebral	13 (13)	12 (15.2)	0.670
Carotid	5 (5)	13 (16.5)	0.011
Vertebral	3 (3)	1 (1.27)	0.430
Renal	11 (11)	6 (7.6)	0.440
Mesenteric	13 (13)	11 (14)	0.857
Iliac	4 (4)	8 (10.1)	0.104
Aorta	7 (7)	25 (31.6)	<0.001
Thoracic	6	24	
Abdominal	1	1	
Other artery dissection^a^^,^^b^, n (%)	12 (12)	37 (46.8)	<0.001
Coronary	4 (4)	5 (6.3)	0.479
Mesenteric	3 (3)	21 (26.6)	<0.001
Iliac	4 (4)	4 (5.1)	0.732
Renal	5 (5)	11 (14)	0.038
Aortic	0	1 (1.3)	0.251
Number of arterial beds with aneurysm, dissection, FMD, n (%)			
1	11 (11)	5 (6.3)	
2	27 (27)	41 (52)	
≥3	62 (62)	33 (41.7)	

Abbreviations as in Table 1.

a Identified on index cross-sectional imaging.

b Locations are not mutually exclusive.

### Recurrent cervical and extracervical artery events

Among 315 patients, the median duration of clinical follow-up was 28 months (Q1, Q3: 11.0, 71.0 months). Recurrent CeAD (Table 3) occurred most frequently in patients with isolated CeAD (27 events; 19.8%), compared with FMD-CeAD (10 events; 10.0%) and MAAD-CeAD (9 events; 11.4%). By contrast, incident extracervical artery dissection occurred most frequently in the MAAD-CeAD group (16 events; 20.2%), compared with FMD-CeAD (3 events; 3.0%), and was not observed in patients with isolated CeAD. Given differences in follow-up duration across phenotypes, these event counts are presented descriptively; time-to-event analyses are shown in Figure 3.

**Table 3:** Rate of Recurrent Arterial Events after Index Head-to-Pelvis Imaging in Patients With CeAD

	Isolated CeAD (n = 136)	FMD-CeAD (n = 100)	MAAD-CeAD (n = 79)
Duration of clinical follow-up (mo), median, (Q1, Q3)	20.5 (9.0, 59.5)	35.5 (16.5, 88.0)	29 (14.0, 64.0)
Recurrent cervical artery dissection, n (%)	27 (19.8)	10 (10)	9 (11.4)
Same artery^a^	8	10	1
New artery^a^	22	1	8
Mean time from original dissection (mo), mean ± SD	34 ± 36.1	39 ± 26	37.6 ± 53.3
Incident extracervical artery dissection, n (%)	0 (0)	3 (3)	16 (20.2)
Coronary^a^		2	2
Mesenteric^a^		1	12
Iliac^a^		1	2
Renal^a^		0	4
Number of arteries involved in subsequent dissection events, n (%)			
1	14	9	8
2	9	4	5
3	0	0	2
4	0	0	1
≥5	0	0	2

Abbreviations as in Table 1.

a Not mutually exclusive.

**Figure 3:**
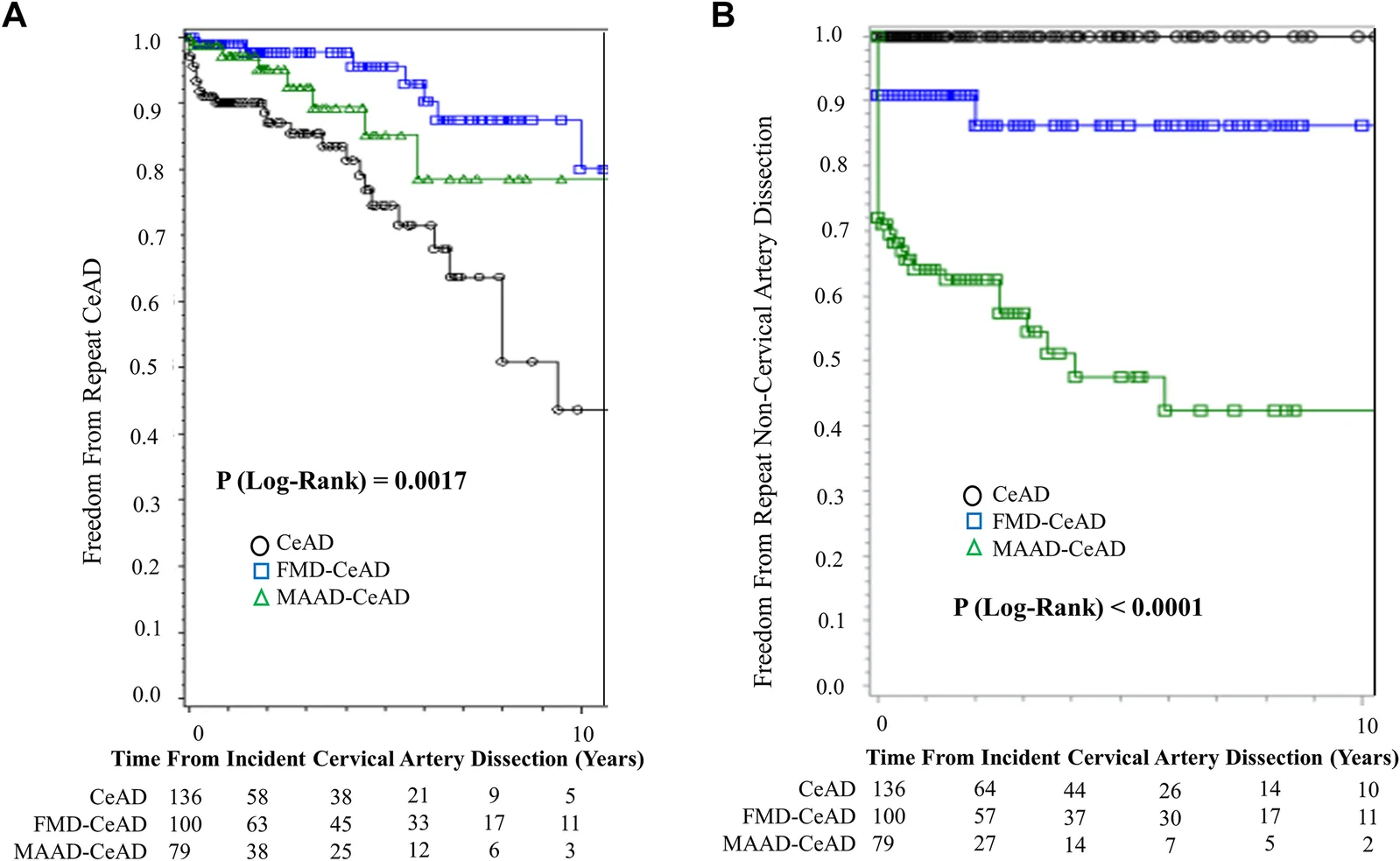
**Freedom From Recurrent Cervical and Extracervical Artery Events by Phenotype** Kaplan-Meier curves representing freedom from (A) repeat cervical artery dissection by phenotype over time: isolated CeAD, FMD-CeAD, and MAAD-CeAD, and (B) incident extracervical artery dissection by phenotype over time: isolated CeAD, FMD-CeAD, and MAAD-CeAD. CeAD = cervical artery dissection; FMD = fibromuscular dysplasia; MAAD = multiple artery aneurysm and dissection.

**Central Illustration:**
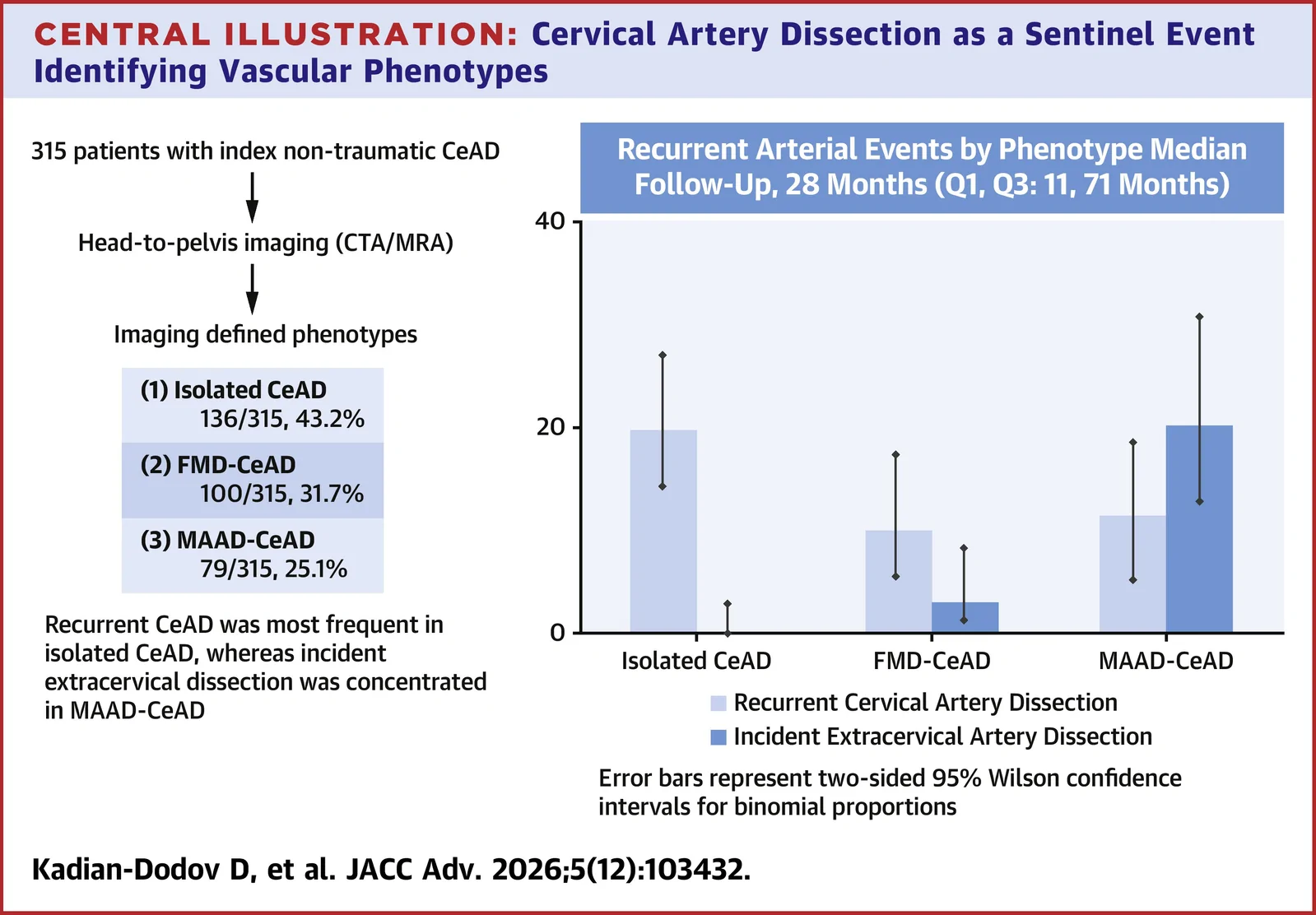
**Cervical Artery Dissection as a Sentinel Event Identifying Vascular Phenotypes** Among patients with nontraumatic cervical artery dissection, head-to-pelvis vascular imaging identified 3 clinically distinct phenotypes: isolated CeAD, FMD-CeAD, and MAAD-CeAD. These phenotypes were associated with different patterns of recurrent arterial events. Recurrent cervical artery dissection was most frequent in isolated CeAD, whereas incident extracervical artery dissection was concentrated in MAAD-CeAD. These findings support a cardiovascular arteriopathy framework for CeAD and suggest that vascular phenotype may inform imaging, surveillance, genetic evaluation, counseling, and longitudinal care. CeAD = cervical artery dissection; CTA = computed tomography angiography; FMD = fibromuscular dysplasia; MAAD = multiple artery aneurysm and dissection; MRA = magnetic resonance angiography.

In multivariable Cox proportional hazards models for recurrent CeAD, adjusted for age at CeAD diagnosis, sex, hypertension, and aneurysm presence, and using FMD-CeAD as the reference group, isolated CeAD was associated with higher recurrence risk (HR: 3.26; 95% CI: 1.43-7.43), whereas MAAD-CeAD was not (HR: 1.77; 95% CI: 0.66-4.75); increasing age at CeAD diagnosis was associated with lower recurrence risk (HR: 0.97; 95% CI: 0.94-0.99 per 1-year increase). Because no incident extracervical artery dissections occurred in the isolated CeAD group, the Firth penalized multivariable analysis of this outcome compared FMD-CeAD with MAAD-CeAD. Among the 179 patients with FMD-CeAD or MAAD-CeAD, 19 experienced an incident extracervical artery dissection during follow-up. In unadjusted Firth penalized Cox regression, the estimated hazard was higher with MAAD-CeAD, although the CI included the null (HR: 2.01; 95% CI: 0.75-5.32; *P* = 0.160). After adjustment for age at the index CeAD, MAAD-CeAD was associated with a higher hazard of incident extracervical artery dissection (HR: 3.75; 95% CI: 1.17-12.00; *P* = 0.026). The increase in effect size after age adjustment suggests confounding by age, as patients with MAAD-CeAD were younger at presentation than patients with FMD-CeAD (mean age, 48.5 vs 52.0 years; *P* = 0.04). The association was materially unchanged after additional adjustment for baseline aneurysm (HR: 3.68; 95% CI: 1.11-12.16; *P* = 0.033).

Among patients with MAAD-CeAD, extracervical artery dissections most often involved the mesenteric and renal arteries, and 50% of affected patients experienced 2 or more new dissections during follow-up.

### Genetic evaluation in selected patients

Genetic testing was performed in a subset of patients (Supplemental Tables 1 to 3). Among those with isolated CeAD, 36 patients underwent testing, with pathogenic variants identified in *COL3A1* (vascular Ehlers-Danlos syndrome), *COL1A1* (osteogenesis imperfecta) and *FBN1* (Marfan syndrome); additional variants were nondiagnostic or of uncertain significance. Among patients with FMD-CeAD, 12 underwent genetic testing, with 1 pathogenic variant identified in the *ELN* gene. In the MAAD-CeAD group, 42 patients (53%) underwent genetic testing, yielding a definitive diagnosis in 7 patients, including pathogenic variants in *COL3A1, SMAD4*, and *SMAD3* consistent with heritable connective tissue disorders (vascular Ehlers-Danlos syndrome, *SMAD4*-related aortopathy with juvenile polyposis syndrome, and Loeys-Dietz syndrome type 3, respectively). Among 17 patients with MAAD-CeAD and a VUS, 5 (29.4%; exact 95% CI: 10.3% to 56.0%) experienced a subsequent arterial dissection during follow-up, including 2 recurrent cervical and 3 incident extracervical dissections. The remaining 18 had negative genetic testing results.

## Discussion

In this single-center cohort of patients with nontraumatic CeAD who underwent comprehensive head-to-pelvis vascular imaging, 57% had systemic vascular findings including FMD, aneurysm, and/or dissection with nearly 40% identified on chest/abdomen/pelvis imaging. Imaging-defined phenotypes demonstrated distinct recurrent-event patterns: isolated CeAD was associated with a higher risk of recurrent CeAD, whereas MAAD-CeAD was associated with a higher hazard of incident extracervical artery dissection compared with FMD-CeAD after adjustment for age at the index CeAD. This estimate was materially unchanged after additional adjustment for baseline aneurysm. Together, these findings suggest that CeAD often occurs in the context of broader arteriopathy and that systematic vascular phenotyping may identify clinically meaningful subgroups with different patterns of subsequent risk.

These findings place CeAD within the broader context of cardiovascular arteriopathies, rather than solely within a cerebrovascular diagnostic framework. The overlap among CeAD, FMD, SCAD, aneurysm/dissection syndromes, and heritable arteriopathies suggest the index CeAD may function as a sentinel event identifying patients that warrant broader vascular assessment. This framework is particularly relevant to cardiovascular clinicians who evaluate patients with aortic disease, SCAD, FMD, renal and mesenteric artery dissection, and unexplained aneurysm/dissection phenotypes.

Prior studies of CeAD and FMD often relied on targeted or incomplete extracervical artery imaging, limiting the ability to classify patients with systemic aneurysm or dissection outside of the cervical arteries.^3^, ^9^, ^10^, ^17^, ^18^, ^19^ By contrast, uniform baseline head-to-pelvis imaging in the present study enabled classification of patients into phenotypes defined not only by the presence of FMD, but also by broader patterns of aneurysm and dissection. This more comprehensive approach may help explain heterogeneity in prior estimates of recurrent risk across CeAD populations.

The present findings have several potential clinical implications. First, the high prevalence of systemic vascular abnormalities identified on baseline imaging suggests that limiting evaluation to the cervical circulation risks missing clinically relevant disease in other arterial beds. Second, the divergent recurrence patterns observed in this cohort suggest that CeAD phenotypes may not simply reflect differences in baseline imaging findings but may also identify different longitudinal risk profiles. Similar findings have been reported in patients with renal artery dissection, highlighting that arterial dissection is often not an isolated event.^20^ Finally, the enrichment of heritable arteriopathies within the MAAD-CeAD phenotype underscores the potential role of targeted genetic evaluation in selected patients, particularly those with multivessel involvement or a family history of vascular disease.

These findings support a phenotype-guided approach to post-CeAD evaluation. Rather than basing imaging decisions only on the index cervical presentation, clinicians may consider whether comprehensive vascular imaging can identify FMD, extracervical aneurysm or dissection, or MAAD features that alter counseling and follow-up. The current manuscript data show no major differences across groups in index CeAD location, pseudoaneurysm, presenting symptoms, or CeAD-related intervention rates in the baseline table, supporting the idea that the index clinical presentation alone may not reliably identify the broader vascular phenotype.

Taken together, these data support consideration of one-time head-to-pelvis imaging after CeAD to define vascular phenotype, refine risk stratification, and inform surveillance strategies, providing a rationale for evaluating CeAD more similarly to established approaches in FMD and SCAD.^11^, ^12^, ^14^

### Study limitations

Several limitations should be considered. This was a single-center cohort from a tertiary vascular medicine practice with expertise in FMD, CeAD, and SCAD, and the prevalence of systemic vascular abnormalities may therefore not be generalizable to all CeAD populations. Follow-up imaging intervals and imaging modality were determined clinically, which may have introduced heterogeneity in detection of asymptomatic events. Because surveillance intensity may have differed according to baseline phenotype and clinical findings, ascertainment of recurrent vascular events may also have varied across groups. In addition, only 19 incident extracervical artery dissections occurred among patients with FMD-CeAD or MAAD-CeAD. Although Firth bias correction and parsimonious modeling were used to reduce small-sample bias, CIs were wide, and the MAAD-CeAD association reached statistical significance only after adjustment for age. These findings should therefore be considered exploratory and require validation in larger cohorts. Finally, genetic testing was not performed systematically across the cohort. The observed frequency of subsequent arterial dissection among MAAD-CeAD patients with a VUS is hypothesis-generating. Because genetic testing was performed in a clinically selected subset, the sample was small, and VUSs are by definition insufficiently evidenced to attribute causality, these data do not establish an association between VUS status and recurrent arterial events.

Despite these limitations, this study provides evidence that comprehensive vascular imaging can identify substantial heterogeneity within CeAD and may help define phenotype subgroups with different patterns of recurrent vascular risk. Prospective multicenter studies will be important to validate these phenotypes, assess generalizability, and determine whether systematic vascular phenotyping can improve risk stratification and longitudinal management after CeAD.

## Conclusions

In this single-center cohort of nontraumatic CeAD, systematic head-to-pelvis imaging identified frequent extracervical vascular abnormalities and distinguished phenotypes associated with different patterns of recurrent vascular events. These findings suggest that CeAD may often reflect a broader arteriopathy rather than an isolated cervical vascular event and support prospective evaluation of systematic vascular phenotyping to refine risk stratification and follow-up after nontraumatic CeAD.

Perspectives **COMPETENCY IN MEDICAL KNOWLEDGE:** Nontraumatic CeAD may represent a sentinel vascular event rather than an isolated cervicocranial disorder. Head-to-pelvis vascular imaging can identify distinct phenotypes, including isolated CeAD, FMD-CeAD, and MAAD-CeAD, that are associated with different recurrent arterial event patterns.

**TRANSLATIONAL OUTLOOK:** Prospective multicenter studies are needed to validate phenotype-specific risk estimates after CeAD, define optimal imaging and surveillance strategies, and determine which patients benefit from genetic evaluation and longitudinal vascular medicine follow-up.

### Declaration of generative AI and AI-assisted technologies in the writing process

Microsoft 365 Copilot, version number 2.20260602.20.0 was used in the preparation of this work to edit for grammar, spelling, and improve sentence structure. The content produced by artificial intelligence was reviewed and edited as needed, and the authors take full responsibility for the accuracy, validity, originality, and final content presented.

## Funding support and author disclosures

Drs Kadian-Dodov, Olin, and Kovacic acknowledge support from the NIH (R01HL148167). Dr Kovacic also acknowledges support from NIH grants R01HL175992, R01HG012773, the Leducq Foundation (25CVD02), and the Bourne Foundation. All other authors have reported that they have no relationships relevant to the contents of this paper to disclose.
