# Supplementary material for: Vascular Phenotypes and Recurrent Arterial Events After Nontraumatic Cervical Artery Dissection
Source: JACC Adv. 2026 Nov 5;5(12):103432. doi: 10.1016/j.jacadv.2026.103432 (PMC13761259; doi:10.1016/j.jacadv.2026.103432)
Supplement: Supplementary file 1 [file mmc1.docx]

**Table S1. Identified Genetic Variants and Clinical Features of Patients That Underwent Genetic Testing With Isolated CeAD**

|  | Patient Features | Gene | Variant | Variant classification | Family History |
| --- | --- | --- | --- | --- | --- |
| 1 | 32 yo male with right internal carotid artery dissection, small pulmonary arteriovenous malformation identified on echocardiogram | *COL3A1*  *CBS* | c.4254G>A, p.T1418=  c.1105C>T, p.R369C | P‡  VUS | Mother with innominate artery dissection following endovascular intervention (68 years) |
| 2 | 40 yo female with left internal carotid artery dissection, history of chiari malformation and tonsillar herniation | *FBN1* | c.7204+3 A>G, IVS58+3 A>G | P‡ | Brother with thoracic aortic aneurysm s/p David Procedure, maternal grandfather deceased from ruptured abdominal aortic aneurysm (Age 60s) |
| 3 | 39 yo male with left internal carotid artery dissection, extreme tortuosity of RICA | *COL1A1*  ZNF469 | c.1862_1865del (p.P621Lfs*144)  c.11492C>T, p.P3831L | P  VUS/LB | Paternal grandfather with fatal MI (Age 60s), paternal uncle with MI 60s |
| 4 | 35 yo male with right internal carotid artery dissection and right vertebral artery dissection. | *FKBP14* | c.362dup (p.Glu122Argfs*7) | P^†^ | Maternal grandfather with sudden cardiac death in 60s |
| 5 | 43 yo female with left internal carotid artery dissection | *COL9A1*  *TGFB3* | c.706C>T, p.Q236*  c.626G>A, p.Arg209His | P^†^  VUS | Maternal aunt with fatal stroke due to ruptured arteriovenous malformation (age unknown), maternal grandfather with ruptured aortic aneurysm (unknown if thoracic or abdominal) |
| 6 | 29 yo female with bilateral vertebral and right internal carotid artery dissection | *COL1A2* | c.286A>G, p.M96V | VUS | Paternal grandfather with stroke age 50 years old |
| 7 | 32 yo male with left vertebral artery dissection | *ACTA2* | c.107 T>C p.I36T | VUS | Father brainstem arteriovenous malformation resulting in fatal stroke age 68 years |
| 8 | 30 yo female with bilateral vertebral artery dissection | *FBN2* | c.472A>T, p.T158S | VUS | Negative |
| 9 | 34 yo male with right internal carotid dissection and right vertebral artery dissection | *FBN*  *NOTCH1* | c.4444G>A, p.G1482S  c.5224G>T, p.A1742S | VUS  VUS/LB | Mother stroke age 60 years, maternal aunt aortic aneurysm (unknown if thoracic or abdominal) |
| 10 | 30 yo female with right vertebral artery dissection | *COL5A2*  *TGFB3* | c.4316T>C, p.Ile1439Thr  c.757G>A, p.Val253Met | VUS  VUS | Negative |
| 11 | 37 yo male with bilateral internal carotid artery dissection, hip hypermobility | *TGFB3* | c.754+5C>T, p.? | VUS/LB | Maternal great uncle and great aunt with sudden cardiac death (Age 70) |
| 12 | 41 yo female with right internal carotid artery dissection, cervical insufficiency during pregnancy requiring cerclage. | *COL5A1* | c.4481C>T, p.P1494L | VUS/LB | Brother died of sudden cardiac death (Age 32) |
| 13 | 32 yo female with right vertebral artery dissection with history of hypermobility | *TNXB* | c. 4010G>A, p.R1337H | N/A | Hypermobility on exam, Beighton = 6 |
| 14 | 33 yo female with left vertebral artery dissection | *AEBP1* | c.2151 G>A, p.W717X | N/A | Maternal grandmother with fatal aortic dissection (Age 46), maternal uncle with spontaneous coronary artery dissection (Age 39) |
| 15 | 32 yo female with left internal carotid artery dissection | *PRKG1* | c.721C>T, p.P241S | VUS | Paternal grandfather with thoracic aortic aneurysm, father with bicuspid aortic valve |
| 16 | 41 yo female with left vertebral artery dissection and aortic root dilation (3.3 cm) | *FLNA* | c.5677 G>T, p. A1893S | N/A | Maternal uncle with sudden cardiac death (Age 52) |
| 17 | 27 yo male with bilateral carotid artery dissection, left vertebral artery dissection, marked cervical artery tortuosity | *COL11A1*  *COL2A1*  *TNXB* | c.904G>A, p.A302T  c.121T>C, p. Y41H  c. 7462 G>C, p.V2488L | VUS  VUS  N/A | Maternal grandmother with sudden death (Age 60) |
| 18 | 27 yo female with left internal carotid artery dissection and recurrent right internal carotid artery dissection at age 36. | *ADAMTS17*  *LTBP4*  *PLOD3* | c.548A>G, p.K183R  c.4051G>A, p.A1351T  c.1966T>C, Y656H | LB  VUS  VUS | Negative |
| 19 | 56 yo male with right internal carotid artery dissection, left vertebral artery dissection, marked cervical artery tortuosity | Negative |  |  | Negative |
| 20 | 23 yo female with right vertebral artery dissection and recurrent bilateral vertebral artery dissection at age 31 years old. | Negative |  |  | Maternal uncle with fatal MI (Age 50s), paternal uncle with fatal MI (Age 50s), grandfather with fatal MI (Age 60s) |
| 21 | 32F yo female with bilateral vertebral artery dissections and recurrent right vertebral artery dissection at age 34. | Negative |  |  | Negative |
| 22 | 26 yo female with left internal carotid artery dissection and recurrent bilateral vertebral artery dissections at age 30. | Negative |  |  | Mother with FMD |
| 23 | 40 yo female with bilateral internal carotid artery dissection and recurrent left vertebral artery dissection 1 month later. | Negative |  |  | Negative |
| 24 | 38 yo female with left internal carotid artery dissection and recurrent left vertebral artery dissection 2 months later. Small uvula on exam. | Negative |  |  | Negative |
| 25 | 55 yo male with right internal carotid artery dissection and recurrent left internal carotid artery dissection 1 day later, bilateral vertebral artery dissections 1 month later | Negative |  |  | Cousin with cerebral aneurysm |
| 26 | 33 yo female with bilateral vertebral artery dissections | Negative |  |  | Negative |
| 27 | 44 yo female with right internal carotid artery dissection and recurrent right vertebral and left internal carotid artery dissection at age 50. | Negative |  |  | Mother with fatal hemorrhagic stroke at age 80. |
| 28 | 35 yo male with right vertebral artery dissection and recurrent bilateral vertebral artery dissections at age 39. | Negative |  |  | Negative |
| 29 | 36 yo female with left internal carotid artery dissection and recurrent right internal carotid artery and left vertebral artery dissection 7 months later. | Negative |  |  | Negative |
| 30 | 48 yo male with bilateral internal carotid and vertebral artery dissection, aortic root dilation 3.6 cm | Negative |  |  | Negative |
| 31 | 23 yo female with bilateral vertebral artery dissection with hypermobility and atrophic scars | Negative |  |  | Negative |
| 32 | 34 yo female with right carotid artery dissection and hEDS. | Negative |  |  | Great grandfather with fatal MI at “young age” |
| 33 | 54 yo female with bilateral internal carotid artery dissection, left vertebral artery dissection | Negative |  |  | Paternal aunt with spontaneous coronary artery dissection and FMD (age 50 years at diagnosis), paternal aunt with cerebral aneurysm and sudden death due to ruptured mesenteric aneurysm (age 40 years), mother with abdominal aortic aneurysm after age 60 years |
| 34 | 38 yo male with left carotid artery dissection | Negative |  |  | Negative |
| 35 | 46 yo female with bilateral vertebral artery dissection and left internal carotid artery dissection. History of hypermobility with joint subluxations, pes planus. | Negative |  |  | Father and paternal grandfather with MI in 40s. |
| 36 | 29 yo female with bilateral vertebral artery dissection, hypermobile EDS | Negative |  |  | Negative |

LB- likely benign; LP- likely pathogenic; P- pathogenic; VUS- variant of uncertain significance

†Heterozygous carrier for autosomal recessive condition

‡Diagnostic genetic testing result

**Table S2. Identified Genetic Variants and Clinical Features of Patients That Underwent Genetic Testing With CeAD-FMD**

|  | Patient Features | Gene | Variant | Variant classification | Family History |
| --- | --- | --- | --- | --- | --- |
| 1 | 48 yo female with left vertebral artery dissection and left internal carotid artery dissection | *ELN*  *PRDM5* | c.1933+ 1G>C, (splice donor)  c.106G>A, p.Gly36Arg | LP†  VUS | Mother with thoracic aortic aneurysm s/p repair, maternal uncle with ruptured aortic aneurysm (Age 49) unclear if thoracic or abdominal, |
| 2 | 68 yo female with left vertebral artery dissection, multiple cerebellar strokes | *FBN1*  *MYLK* | c.8071G>A (p.G2691S)  c.4268C>T (p.Thr1423Met) | LB/VUS  VUS | Negative |
| 3 | 4 yo female with right vertebral artery dissection with cerebral aneurysm and ectasia of bilateral internal carotid arteries, basilar artery, bilateral renal arteries and common iliac arteries. | *COL5A2* | c. 4085A>G, p. (Y1382C) | LB/VUS | Negative |
| 4 | 46 yo female with left vertebral artery dissection, splenic artery aneurysm | *COL5A1*  *ZNF469* | c.2578C>T, p.P860S  c.10721C>T, p. P3574L | VUS  VUS | Sister with stroke in basal ganglia (Age 50s) |
| 5 | 33 yo female with right vertebral artery dissection and subsequent left vertebral artery dissection at age 39. | *TGFBR1* | c.1433A>G, p.N478S | LB/VUS | Father with thoracic aortic aneurysm and dissection (Age 61), paternal aunt with bowel rupture, paternal uncle with bowel rupture (details not known) |
| 6 | 38 yo female with history of cerebral aneurysms s/p clipping, left internal carotid artery and right vertebral artery dissections and subsequent spontaneous coronary artery dissection, mesenteric artery dissection | Negative |  |  | Daughter with Chiari malformation |
| 7 | 57 yo female with right vertebral artery dissection, cervical artery and iliac artery tortuosity | Negative |  |  | Multiple family members with hypermobility, brother with cardiac event age 61 years |
| 8 | 47 yo female with right internal carotid artery dissection and subsequent left internal carotid artery dissection at age 51, retinal detachment, right cerebral aneurysm | Negative |  |  | Negative |
| 9 | 55 yo female with left internal carotid artery dissection and left vertebral artery aneurysm | Negative |  |  | Negative |
| 10 | 50 yo female with history of splenic artery aneurysm coiling, left internal carotid artery dissection and left renal artery aneurysm | Negative |  |  | Daughter hypermobile |
| 11 | 50 yo female with vertebral artery dissection, splenic artery aneurysm and celiac artery aneurysm | Negative |  |  | Aortic valve replacement in brother and father |
| 12 | 29 yo female with right vertebral artery dissection and subsequent left vertebral artery dissection and bilateral carotid dissections at age 39. | Negative |  |  | Negative |

LB- likely benign; LP- likely pathogenic; VUS- variant of uncertain significance

†Although primary association is with autosomal dominant supravalvular aortic stenosis and autosomal dominant cutis laxa, the *ELN* gene has also been associated with aortic root dilatation and other connective tissue features (although no reported association with arterial dissection)

**Table S3. Identified Genetic Variants and Clinical Features of Patients That Underwent Genetic Testing With MAAD-CeAD**

|  | Patient Features | Gene | Variant | Variant classification | Family History |
| --- | --- | --- | --- | --- | --- |
| 1 | 40 yo female with left vertebral artery dissection and pseudoaneurysm, celiac artery aneurysm | *COL3A1* | c.2959G>A, p.Gly987Ser | P^‡^ | Father with thoracic aortic aneurysm, paternal aunt with fatal thoracic aortic dissection (Age 70), paternal grandmother with fatal abdominal aortic dissection (Age 70) |
| 2 | 36 yo male with right internal carotid artery dissection and left A2 aneurysm (2mm), prior thoracic aortic aneurysm at age 35 years s/p valve sparing aortic root replacement. | *SMAD4*  *MYH11*  PLOD1 | c.1017dup; p.Lys340*  c.452C>T, p.Pro151Leu  c.608G>A, p.Arg203His | P^‡^  VUS  LB/VUS | Negative |
| 3 | 42 yo female with bilateral vertebral artery dissection resulting in pseudoaneurysm and occlusion, right iliac artery dissection | *COL3A1* | c.709G>A, p.Gly237Arg | P^‡^ | Mother and maternal cousin with fatal type A aortic dissection. Maternal great aunt with sudden death (Age 50s) thought to be due to MI. |
| 4 | 33 yo female with bilateral vertebral artery dissection, left internal carotid aneurysm, and thoracic aortic aneurysm (5.1 cm) with contained rupture that occurred during pregnancy, bicuspid aortic valve s/p valve sparing aortic root replacement | *SMAD3* | deletion (exon 2-9) | P^‡^ | Father with fatal aortic dissection (Age 40s) |
| 5 | 36 yo male with left internal carotid artery dissection, bilateral carotid artery tortuosity, thoracic aorta (4.1 cm), ectasia or abdominal aorta (2.4 cm), dissection and aneurysm of right common iliac artery (2.4 cm), left iliac artery ectasia (1.7 cm) and internal iliac artery (1.3 cm). Subsequent right internal carotid artery dissection 5 years later. | *COL3A1* | c.3436 G>T, p. G1146* | P^‡^ | Maternal grandfather’s father stroke (Age 40s), maternal great- uncle sudden death (Age 40s), maternal uncle died in infancy. |
| 6 | 49 yo female with right vertebral artery dissection, bilateral renal artery dissection resulting in infarcts. Subsequent bilateral carotid artery dissection 14 years later, splenic artery aneurysms infrarenal aorta ectasia, celiac artery ectasia. | *COL3A1* | c.1905del, p.Gly636Valfs*155 | P^‡^ | Brother fatal splenic artery rupture (Age 54), Brother sudden cardiac arrest (Age 68). |
| 7 | 38 yo female with bilateral carotid artery dissection and left pseudoaneurysm, vertebral artery tortuosity. Subsequent bilateral renal artery dissection 5 years later, celiac and right renal artery dissections 7 years later, and splenic artery dissection with pseudoaneurysm 8 years later. | COL3A1 | c.785_786insTTT, p.Met262delinslleLeu | LP^‡^ | Mother with bladder/uterine prolapse requiring surgery, maternal uncle and maternal grandmother died in 60s due to presumed MI |
| 8 | 22 yo male with left vertebral artery dissection and thoracic aorta (3.7 cm) at sinus of valsalva | *FBN2*  *NOTCH1* | c.559G>A, p.Gly187Ser  c.2535G>A, p.Gly785Ser | LB  VUS | Negative |
| 9 | 38 yo female with left vertebral artery dissection | *LTBP4* | c.4468G>A, p.Glu1490Lys | VUS | Negative |
| 10 | 49 yo female with left internal carotid artery dissection and right internal carotid artery aneurysm s/p repair, SMA ectasia and tortuosity of the descending thoracic and abdominal aorta. | *FBN2* | c.2342A>G, p.N781S | LB/VUS | Negative |
| 11 | 38 yo female with left vertebral artery dissection and subsequent celiac artery dissection extending into the hepatic artery | Chromosomal abnormality^⨍^ | 405 kb gain (duplication) of 22q11.21 | VUS^⯎^ | Paternal uncle cardiac arrest (Age 69) |
| 12 | 58 yo female with right internal carotid artery, right vertebral artery dissection, left renal artery dissection | *COL3A1* | c.1978-9A>G, intronic | VUS | Brother fatal aortic dissection (Age 47), maternal uncle with sudden death due to presumed MI (Age 52) with h/o substance use |
| 13 | 27 yo male with bilateral vertebral artery dissection. Subsequent spontaneous coronary artery dissection 12 years later, celiac artery dissection and pseudoaneurysm, right CIA dissection with pseudoaneurysm and bilateral external iliac artery dissection, right internal iliac artery dissection | *ACTA2* | c.295 C>G, p.A99G | VUS | Negative |
| 14 | 32 yo female with bilateral vertebral artery dissections, right renal artery dissection | *BGN* | c.181G>A, p.Ala61Thr | B/LB/VUS | Negative |
| 15 | 69 yo male with left internal carotid artery dissection resulting in hemispheric stroke, subsequent SMA dissection resulting in mesenteric ischemia 10 months later. | *B3GALT6* | c.604G>A, p.A202T | VUS | Negative |
| 16 | 48 yo male with right internal carotid artery dissection and celiac artery dissection with pseudoaneurysm, vertebral artery tortuosity and common iliac artery tortuosity. | *COL2A1*  *MYLK*  *PRKG1* | c.3977C>T, p.P1326L  c.4291C>T, p.P1431S  c.389 T>C, p.I130T | VUS  VUS  VUS | Father with aortic dissection (age 74), two paternal uncles with abdominal aortic aneurysm rupture (Age 50s) |
| 17 | 57 yo male with left vertebral artery dissection with prior ST-elevation myocardial infarction that may have been spontaneous coronary artery dissection | *MYH11* | c.505 C>T, p.R169W | VUS | Negative |
| 18 | 48 yo male with bilateral carotid artery dissections, bilateral vertebral artery dissections with areas of pseudoaneurysm resulting in neck swelling at presentation requiring intubation for airway protection; renal infarct on imaging through to be due to dissection. | *PLOD1* | c.1550 A>C, p.H517P | VUS | Negative |
| 19 | 69 yo female with bilateral carotid dissection, thoracic aorta (4.1 cm), tortuosity of carotid, aorta and iliac arteries | *COL11A2*  *COL9A1* | c.1220C>T, p.A407V  c.2159G>A, p.R720Q | VUS  LB/VUS | Daughter with shoulder dislocations. |
| 20 | 64 yo male with bilateral carotid artery dissection with left vertebral artery dissection, splenic artery aneurysm, two SMA aneurysms, common iliac artery aneurysm, internal iliac artery aneurysm. | *FBN2* | c.7007G>A, p.Cys2336Tyr | LB/VUS | Maternal uncle with fatal MI (Age 40s), sister with mitral valve prolapse, daughter with hypermobility |
| 21 | 58 yo male with right internal carotid artery dissection resulting in ischemic stroke, internal carotid artery and bilateral vertebral artery tortuosity. Dissection of celiac and SMA with pseudoaneurysm and iliac artery aneurysm. | *MYLK*  *SKI* | c.4838-3C>T (intronic)  c.1321C>T, p.Arg441Trp | LB/VUS  LB/VUS | Mother and maternal grandmother with fatal MI (age late 40s). |
| 22 | Left internal carotid artery dissection and subsequent left vertebral artery dissection 2 months later. Dilated aortic root (3.8 cm), and hypermobile EDS with slipped rib syndrome requiring surgeries, retinal tears, dysautonomia. | *COL5A2*  *MYLK* | c.379A>C, p.Ile127Leu  c.1802A>G, p.His601Arg | VUS  VUS | Father with fatal MI (Age 52), children with hypermobile EDS |
| 23 | 46 yo female with bilateral carotid artery dissections, right vertebral artery dissection, and left common iliac artery aneurysm, tortuosity of the infrarenal aorta and iliac arteries. | *COL3A1* | c.226A>G, p.Asn76Asp | B/VUS | Maternal aunt with fatal rupture of cerebral aneurysm (Age 40s) |
| 24 | 46 yo female with RICA dissection and subsequent left internal carotid artery dissection 6 months later, and left vertebral artery dissection 7 years later. Aortic root dilation (3.8 cm) and hypermobile EDS. | *TNXB*  *ADAMTS2* | c.2083C>T, p.R695W  c.1793G>A, p.R598H | VUS  VUS | Paternal grandfather with sudden death due to presumed MI (age 58), maternal uncle with fatal MI, father with abdominal aortic aneurysm (Age 71, smoker) |
| 25 | 42 yo female with left internal carotid artery dissection and left vertebral artery dissection, aortic root enlargement 3.9 cm, Hypermobility (Beighton 6, +thumb in wrist sign) | Negative |  |  | Negative |
| 26 | 65 yo male with bilateral carotid dissection and thoracic aorta (4 cm) at sinus of valsalva, left common and internal iliac artery ectasia. | Negative |  |  | Father and paternal uncles with MI (Age 50s) |
| 27 | 43 yo male with right vertebral artery dissection resulting in PICA stroke s/p tPA complicated by suboccipital craniectomy and thoracic aorta (4 cm), tortuosity and ectasia of the splenic artery (7 mm) and iliac artery tortuosity. | Negative |  |  | Not known, adopted. |
| 28 | 37 yo male with bilateral vertebral artery dissections, ascending tubular aorta 4.2 cm, multiple small cerebral outpouchings (paraclinoid internal carotid artery, anterior communicating artery, left internal carotid artery terminus) | Negative |  |  | Negative |
| 29 | 61 yo male with right internal carotid dissection and large pseudoaneurysm, left carotid tortuosity and aortic root dilation (4.2 cm) | Negative |  |  | Father with mitral valve prolapse |
| 30 | 39 yo female with right vertebral artery dissection, right internal carotid artery tortuosity and right cavernous carotid artery outpouching | Negative |  |  | Negative |
| 31 | 32 yo female with left internal carotid artery dissection and spontaneous coronary artery dissection of circumflex artery | Negative |  |  | Negative |
| 32 | 49 yo female with right vertebral artery dissection and bilateral carotid artery dissection with subsequent celiac and SMA dissection with pseudoaneurysm (Age 50) and iliac artery ectasia | Negative |  |  | Male cousin with sudden death (Age 32) |
| 33 | 52 yo male with bilateral carotid artery dissection, right internal carotid cavernous aneurysm (2.6 mm), thoracic aorta (4.3 cm) and iliac artery tortuosity. | Negative |  |  | Sudden death in father due to ruptured coronary artery aneurysm (age 77) |
| 34 | 55 yo female with bilateral carotid artery dissection, celiac artery dissection and renal artery dissection | Negative |  |  | Paternal grandfather with sudden death (Age 30-40) thought to be due to MI, Maternal grandfather with MI |
| 35 | 60 yo male with right vertebral artery dissection resulting in stroke, left renal artery dissection and aneurysm. Carotid artery tortuosity. | Negative |  |  | Negative |
| 36 | 39 yo female with left internal carotid artery dissection and subsequent right internal carotid artery and left vertebral artery dissection 4 years later. Ascending aorta dilated (3.4 cm), right renal artery aneurysm) | Negative |  |  |  |
| 37 | 43 yo female with right internal carotid artery dissection with subsequent left vertebral artery dissection 1 month later, and left internal carotid artery dissection 3 years later. Pseudoaneurysms of carotid dissection requiring coiling. | Negative |  |  | MGaternal grandfather with stroke (Age 50s), Maternal grandmother with MI (Age 68 years). |
| 38 | 36 yo male with left internal carotid artery dissection and subsequent SMA dissection 12 years later. | Negative |  |  | Negative |
| 39 | 51 yo male with bilateral carotid artery dissection, left common iliac artery dissection, thoracic aorta (4 cm). Subsequent right internal carotid artery dissection 4 years later. | Negative |  |  | Negative |
| 40 | 47 yo male with left vertebral artery dissection, bilateral renal artery dissection, celiac-hepatic-splenic artery dissection, aneurysm of pancreaticoduodenal artery, thoracic aorta (4.3 cm) at sinus of valsalva | Negative |  |  | Negative |
| 41 | 64 yo female with bilateral carotid artery dissection, right vertebral artery dissection, celiac artery dissection with pseudoaneurysm (1.2 cm) extending into hepatic artery, SMA branch dissection with pseudoaneurysm (1 cm). | Negative |  |  | mother (cavernous carotid aneurysm), maternal aunt (cerebral aneurysm), sister (multiple cerebral aneurysms), brother with aortic aneurysm (unclear if thoracic or abdominal) |
| 42 | 58 yo female with right thalamic bleed due to cavernoma s/p resection, left internal carotid artery dissection, splenic artery aneurysm, ascending aorta dilation (4 cm). Subsequent bilateral vertebral artery dissection 2 years later, and new celiac artery dissection 3 years. | Negative |  |  | Negative |

B- benign; LB- likely benign; LP- likely pathogenic; P- pathogenic; VUS- variant of uncertain significance

†Heterozygous carrier for autosomal recessive condition

‡Diagnostic genetic testing result

⯎ Genomic gain in region associated with 22q11.2 duplication syndrome but the phenotypic consequences of duplication are not the same as the common deletion syndrome.

⨍ Negative hTAAD panel testing
